# Incidentally Discovered Splenic Infarction Associated with Scrub Typhus

**DOI:** 10.4269/ajtmh.15-0140

**Published:** 2015-09-02

**Authors:** Jeong-Hwan Hwang, Chang-Seop Lee

**Affiliations:** Department of Internal Medicine, Chonbuk National University Medical School, Jeonju, Republic of Korea; Research Institute of Clinical Medicine, Chonbuk National University-Chonbuk National University Hospital, Jeonju, Republic of Korea

**Figure 1.**
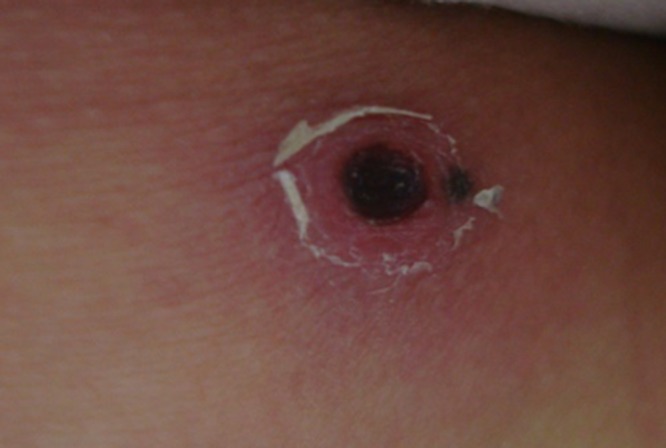
Eschar on the posterior aspect of the right thigh.

**Figure 2.**
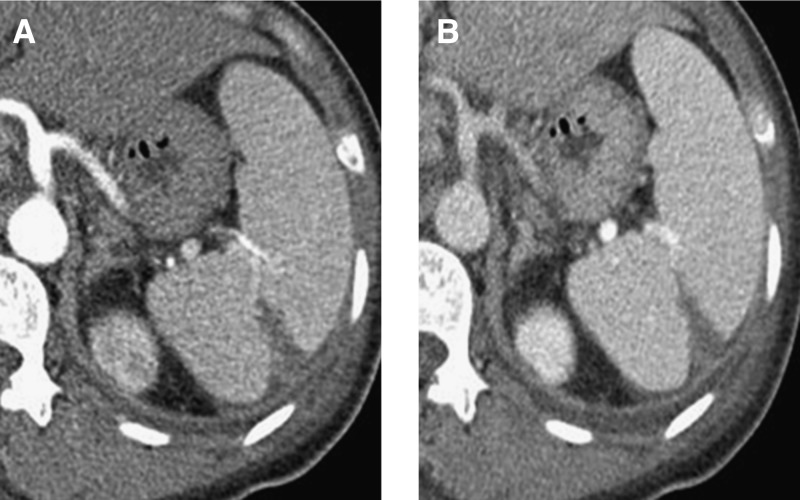
Abdominal computed tomography with enhancement shows a low-attenuated, wedge-shaped lesion consistent with splenic infarction as well as splenomegaly in the arterial phase (**A**) and portal phase (**B**).

A 46-year-old woman was transferred to our emergency room (ER) from another hospital because of pleural effusion and general weakness. Five days before admission to our ER, oral doxycycline treatment was begun on clinical suspicion of scrub typhus. On physical examination by emergency medical services personnel, the patient did not exhibit a skin rash, and eschar was observed on the right posterior thigh aspect (Figure 1

). No abdominal tenderness was observed. On arrival to our ER, transthoracic echocardiography was performed because of both pleural effusion and general weakness, and the results were within normal limits. On laboratory testing, complete blood count was within normal range, as were other chemical profiles except for the following: alkaline phosphatase 229 IU/L, gamma-glutamyl transferase 153 IU/L, aspartate aminotransferase 110 IU/L, and alanine aminotransferase 190 IU/L. Peripheral blood smear showed normal finding. Coagulopathy testing did not reveal disseminated intravascular coagulation, with negative results for lupus anticoagulant and anticardiolipin antibody. Serology for Epstein–Barr virus was also negative. The *Orientia tsutsugamushi* antibody titer using the indirect immunofluorescent antibody test was 1:5,120. Abdominal computed tomography was performed because of abnormal liver function test results, which revealed a wedge-shaped hypoperfusion area, suggesting splenic infarction (Figure 2

). During hospitalization, blood culture did not yield any organism. Oral doxycycline was maintained for more than 7 days. The patient's clinical course improved, and 2 weeks after discharge, the patient was stable without abdominal tenderness.

Focal or disseminated vasculitis is the major pathogenesis of complications following scrub typhus.^1^ Scrub typhus can lead to the involvement of various intra-abdominal organs.^1^ However, the pathogenesis of splenic infarction associated with scrub typhus has not been previously documented, and splenic complications such as splenic infarction may be extremely rare and underreported.^1^,^2^
